# NeRF-OR: neural radiance fields for operating room scene reconstruction from sparse-view RGB-D videos

**DOI:** 10.1007/s11548-024-03261-5

**Published:** 2024-09-13

**Authors:** Beerend G. A. Gerats, Jelmer M. Wolterink, Ivo A. M. J. Broeders

**Affiliations:** 1 AI & Data Science Center, Meander Medical Center, Amersfoort, The Netherlands; 2 Department of Applied Mathematics and Technical Medical Center, University of Twente, Enschede, The Netherlands; 3 Department of Robotics and Mechatronics, University of Twente, Enschede, The Netherlands

**Keywords:** Neural radiance fields, 3D scene reconstruction, Operating room videos, RGB-D imaging, Dense depth estimation

## Abstract

**Purpose:**

RGB-D cameras in the operating room (OR) provide synchronized views of complex surgical scenes. Assimilation of this multi-view data into a unified representation allows for downstream tasks such as object detection and tracking, pose estimation, and action recognition. Neural radiance fields (NeRFs) can provide *continuous* representations of complex scenes with limited memory footprint. However, existing NeRF methods perform poorly in real-world OR settings, where a small set of cameras capture the room from entirely different vantage points. In this work, we propose NeRF-OR, a method for 3D reconstruction of dynamic surgical scenes in the OR.

**Methods:**

Where other methods for sparse-view datasets use either time-of-flight sensor depth or dense depth estimated from color images, NeRF-OR uses a combination of both. The depth estimations mitigate the missing values that occur in sensor depth images due to reflective materials and object boundaries. We propose to supervise with surface normals calculated from the estimated depths, because these are largely scale invariant.

**Results:**

We fit NeRF-OR to static surgical scenes in the 4D-OR dataset and show that its representations are geometrically accurate, where state of the art collapses to sub-optimal solutions. Compared to earlier work, NeRF-OR grasps fine scene details while training 30$\times$ faster. Additionally, NeRF-OR can capture whole-surgery videos while synthesizing views at intermediate time values with an average PSNR of 24.86 dB. Last, we find that our approach has merit in sparse-view settings beyond those in the OR, by benchmarking on the NVS-RGBD dataset that contains as few as three training views. NeRF-OR synthesizes images with a PSNR of 26.72 dB, a 1.7% improvement over state of the art.

**Conclusion:**

Our results show that NeRF-OR allows for novel view synthesis with videos captured by a small number of cameras with entirely different vantage points, which is the typical camera setting in the OR. Code is available via: github.com/Beerend/NeRF-OR.

**Supplementary Information:**

The online version contains supplementary material available at 10.1007/s11548-024-03261-5.

## Introduction

Operating room (OR) videos show relevant information for improving the quality of surgical procedures or increasing safety for patients and medical staff [1, 2]. Over the past decade, many algorithms have been proposed for the automated analysis of these videos [3]. Typically, OR videos are captured simultaneously by multiple cameras that are attached to the wall or ceiling throughout the OR and are directed to the room center [4–6]. The video streams are often accompanied by time-of-flight (ToF) depth measurements such that pixels can be projected over the z-axis. As a result, *discrete* and memory-heavy data formats, such as point clouds and volumetric data grids, are used for video processing [7–10].

**Fig. 1:**
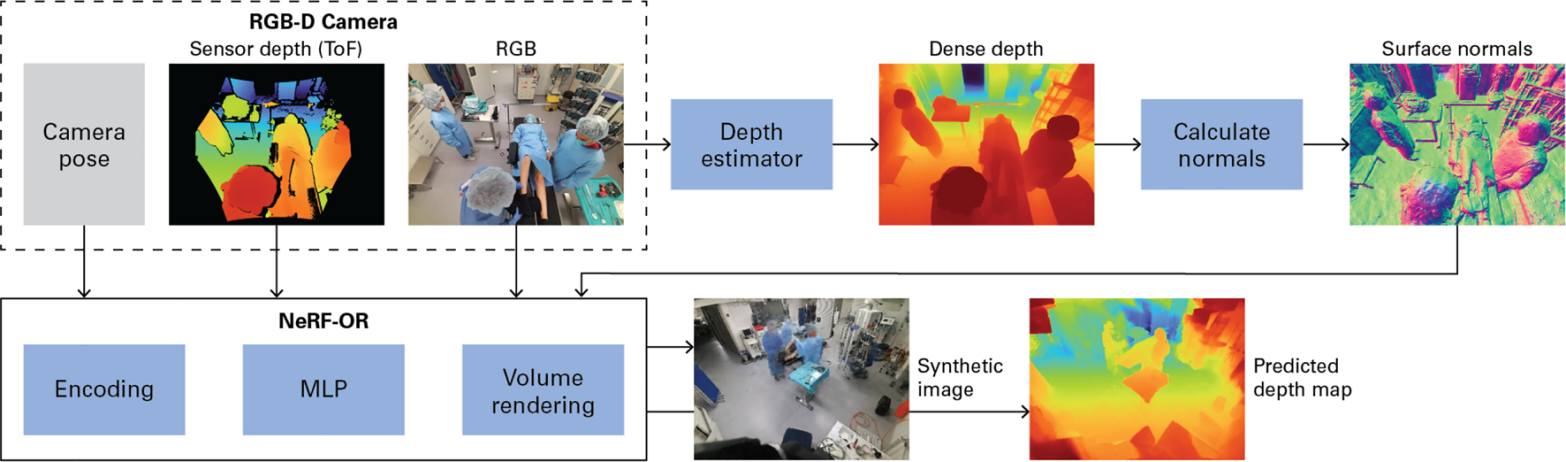
Proposed NeRF-OR approach to build 3D reconstructions of scenes in the operating room. The dataset contains externally calibrated RGB-D cameras. Camera poses, time-of-flight (ToF) sensor depths, and RGB images are provided directly to NeRF-OR. The RGB images are processed by a depth estimator as well, which outputs dense depth maps. We calculate the surface normals from these maps and give the output to NeRF-OR. The method synthesizes images from any new camera position accompanied with predicted depth maps

Recently, neural radiance fields (NeRF) [11] have been adopted as a representation for 3D scenes. In contrast to traditional data formats, NeRF is a *continuous* format with limited memory footprint. The technique enables high-resolution novel view synthesis and allows for the rendering of interactive videos for educational and virtual reality training purposes. Additionally, NeRF representations could be used for many downstream video processing tasks, including pixel tracking [12], mesh rendering [13], point cloud upsampling [14], image deblurring [15], and 3D object detection and segmentation [16, 17].

Although OR video analysis could benefit from NeRF representations, the typical camera setup in the OR limits the applicability of existing NeRF methods. First, the number of cameras used is often limited to a handful of viewpoints, as it is undesirable to place tens of cameras in a sterile environment. Second, the cameras are positioned throughout the OR with entirely different vantage points to mitigate the many occlusions. However, under these conditions, the projection from 2D to 3D is heavily ill-posed.

Various methods have been developed for few-camera (“sparse-view”) datasets, commonly involving three to six input views. RegNeRF [18] uses additional regularization with a smoothness loss and an appearance loss at patches sampled from arbitrary unobserved viewpoints. Deng et al. [19] propose to supervise NeRF reconstructions with depth from sparse 3D points gained during structure-from-motion (SfM) calibration. In our earlier work [20], we showed that supervision with ToF-measured depth is a more successful approach for OR video datasets. However, these sensor depth maps are often not complete, i.e., they contain missing values due to divergent sensor borders, reflective materials, and “depth shadows” around object boundaries. Alternatively, methods use dense depth maps estimated from color images [21, 22]. The difference between these depth image modalities can be seen in Fig. 1. Although dense depth guarantees values for all pixels, the scale of its values is not necessarily correct. For example, depth estimator *Marigold* [23] outputs values between 0 and 1. Wang et al. [22] propose to use a scale-invariant depth ranking loss instead. However, as we show in this paper, this approach collapses to sub-optimal solutions when applied to OR videos.

In this work, we present NeRF-OR, a novel method for 3D scene reconstruction from sparse-view RGB-D videos. In contrast to previously proposed methods, which use either ToF sensor depth or dense depth estimation from color images, we use a combination of both. The key idea is that supervision with sensor depth provides a good starting point for creating the 3D representations and that an additional loss based on dense depth estimations makes it possible to relate pixels with missing sensor depth values to areas where values are available. Because this relation is relative, we use the supervision of surface normals that are calculated from the estimated depths. Surface normals are largely invariant to depth scale and provide information about the relative depth difference between neighboring pixels.

Our contributions are as follows. First, we combine sensor depth and dense depth estimations as model input, which has, to the best of our knowledge, not been proposed before. We show that the integration of estimated depth benefits training with ToF images that contain areas with missing depth values. Second, we use hash encoding to significantly speed up training by a factor of 30 compared to our previous work [20]. Third, we perform an extensive analysis of our method, including a temporal evaluation of dynamic reconstructions from whole-procedure videos. Finally, we assess our method on a sparse-view benchmark and find that it outperforms the state of the art, which shows that our method generalizes to sparse-view camera setups beyond the OR as well.

## Methods

### Neural radiance fields

Neural radiance fields (NeRF) are a method for rendering synthetic images of a volumetric scene from arbitrary camera angles [11]. It uses a multilayer perceptron (MLP) $F_\Theta$ that represents the scene. A dynamic NeRF can render videos from a scene that changes over time. Training a dynamic NeRF requires a set of images that capture the scene at different time values *t* and viewing directions *d*, together with the intrinsic and extrinsic parameters of the cameras used to obtain these images. To optimize a NeRF, camera rays *r* are cast through the scene originating from randomly selected image pixels. A set of spatial 3D locations $\{x_1, x_2, \dotsc , x_S\}$ is sampled along each ray. The MLP is queried with the locations, time values, and viewing directions and returns a material density $\sigma$ and color *c* for each sample:

1 $$\begin{aligned} (c, \sigma ) = F_\Theta (\gamma (x, t), d), \end{aligned}$$

where $\gamma$ is the positional encoding function for locations and time values before they are given to the MLP. Thereafter, the material densities and colors are accumulated along the ray using quadrature resulting in a predicted pixel color:

2 $$\begin{aligned} \hat{C}(r) = \sum ^S_{i=1} U_i (1 - \text {exp}(-\sigma _i \delta _i)) c_i \end{aligned}$$

where $U_i = \text {exp}\big (-\sum ^{i-1}_{j=1}\sigma _j\delta _j\big )$, and $\delta _i$ is the distance between the sampled points along a ray. The predicted colors are compared with the actual colors in the training images:

3 $$\begin{aligned} L_{\text {color}} = \sum _{r \in R} \Vert \hat{C}(r) - C(r) \Vert _2^2 \end{aligned}$$

Subsequently, the error is back-propagated to optimize the MLP, and to the encoding function as well if learnable weights are involved.

**Fig. 2:**
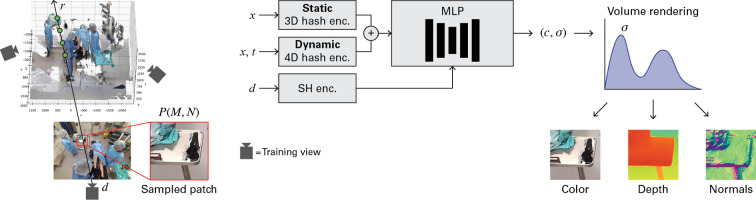
Inner workings of NeRF-OR. Patches of size (*M*, *N*) are randomly sampled from training views with viewing angle *d*. A ray *r* is cast from each pixel in the patch through the 3D scene. Points *x* are sampled along the ray and embedded with hash encoding, together with time value *t*. NeRF-OR uses element-wise addition of static and dynamic encoding. An MLP returns color *c* and material density $\sigma$, used for volume rendering. The method outputs predicted color, depth, and surface normals

### NeRF-OR

We adapt the original NeRF architecture such that it learns geometrically accurate representations of scenes with sparse training views that have very different vantage points. The most prominent adaptations can be seen in Fig. 1, where the method requires additional input in terms of ToF sensor depth and surface normals that are calculated from dense depth. An overview of how NeRF-OR works internally is given in Fig. 2. During training, patches are randomly sampled from training views at time step $t \in \{1,2,\dotsc ,T\}$. For each pixel in the patch, a ray *r* is cast through the virtual scene along which *S* points are sampled: $\{x_1,x_2,\dotsc ,x_S\}$. To speed up the training of the model, we use hash encoding [24], where spatial locations are encoded with a 3D hash grid and spatiotemporal locations are encoded with a 4D hash grid. Camera viewing angle *d* is encoded with spherical harmonics (SH). The MLP learns a function that outputs colors and material densities. These are used during volumetric rendering to provide a predicted color for each pixel. Contrary to the original NeRF design, NeRF-OR outputs predicted depth maps and surface normals as well.

#### Sensor depth supervision

Similar to our earlier work [20], we use an additional loss function where predicted depth $\hat{D}(r)$ is compared to ToF depth measurement $D_{\text {sensor}}(r)$:

4 $$\begin{aligned} \hat{D}(r)= &   \sum ^S_{i=1} U_i (1 - \text {exp}(-\sigma _i \delta _i)) a_i \end{aligned}$$

5 $$\begin{aligned} L_{\text {depth}}= &   \sum _{r \in R} ( \hat{D}(r) - D_{\text {sensor}}(r) )^2 \end{aligned}$$

where $a_i$ is the distance between sampled point *i* and the camera. Note that these distances should have the same metric as the sensor depth, e.g., millimeters, and the depth images should be transformed from the viewpoint of the sensor to the viewpoint of the RGB camera.

#### Surface normals regularization

A disadvantage of ToF depth is that its images are incomplete, i.e., they display zero values for pixels outside the sensor border, at reflective materials, and around object boundaries. Such missing values can be seen in the example depth image in Fig. 4, e.g., at the table in the upper-left corner. As an alternative, it is possible to use dense depth derived from color images with monocular depth estimators. Recently, several of these estimators were proposed that achieve good results [23, 25, 26]. In contrast to ToF depth, these images guarantee values for all pixels, while their values are relatively smooth and particularly well represented at object boundaries. However, in contrast to ToF depth, dense depth estimation does not provide absolute depth values. The scale of depth is relative and is not related to the camera coordinate system. Typically, these depth values are between 0 and 1 [23]. Therefore, we propose to use a combination of measured ToF depth and depths estimated from RGB images. Depth values from ToF result in material density that is correctly positioned in the 3D scene, while estimated depth helps to find the relative depths for areas where no sensor depth is available. Because we are interested in the relative change of depth, we use surface normals calculated from the estimated depth [21]. These normals represent the direction of change, which is distinctive at object boundaries and smooth at surfaces. We compute the surface normals *N*(*r*) by normalizing the depth gradient in the horizontal and vertical directions to a unit vector and construct the loss function as follows:

6 $$\begin{aligned}  &   N(r) = \dfrac{\nabla D(r)}{\Vert ( \nabla _x D(r), \nabla _y D(r), 1 )\Vert _2} \end{aligned}$$

7 $$\begin{aligned}  &   L_{\text {normal}} = \sum _{r \in R} \Vert \hat{N}(r) - N(r) \Vert ^2 \end{aligned}$$

In Eq. 6, *D* is the depth map estimated from a color image. For the final loss, we combine the three losses with weighting factors $\alpha$ and $\beta$:

8 $$\begin{aligned} L = L_{\text {color}} + \alpha L_{\text {depth}} + \beta L_{\text {normal}} \end{aligned}$$

#### Patch-based training

To enable supervision with surface normals, we shift from standard ray-based to patch-based training. Instead of selecting individual rays at random, we select random patches *P* of size (*M*, *N*). We sample all rays $r_{ij}$ in the patch, where $i \in \{1, 2, \dotsc , M\}$ and $j \in \{1, 2, \dotsc , N\}$, and let NeRF-OR return a color, depth, and surface normal for each pixel in the patch. An illustration of this approach is given in Fig. 2. To supervise surface normals at different scales, we sample the patches at $P_{\text {levels}}$ multiple resolutions. At each scale, the patch is sampled with a stride of $2^l$ with $l \in \{0, 1, \dotsc , P_{\text {levels}}-1\}$.

#### 4D hash encoding for dynamic scenes

A drawback of building dynamic NeRF representations are the generally large training times, requiring up to 1300 GPU hours for a video of 300 frames [27]. To accelerate optimization, we adopt hash encoding from Müller et al. [24]. We extend this approach to encode spatiotemporal positions instead of spatial positions only. Similar to the work from Park et al. [28], NeRF-OR creates a static feature embedding using 3D hash encoding while constructing a dynamic feature embedding using 4D hash encoding. In the latter encoding method, the hash grid is 4-dimensional. We combine the two such that static scene elements are encoded with a 3D hash grid since they do not move over time, while the dynamic elements are encoded with a 4D hash grid. Encoding all material with a 4D hash grid would take a lot of unnecessary memory. Importantly, the maximum resolution of the grid ($N_{\text {max}}$) is different in the spatial dimensions than for the temporal dimension, where we always choose the video length *T* as the maximum resolution. To get the final embeddings, we add the static and dynamic feature embeddings element-wise, as we found empirically no added value in the concatenation of the two:

9 $$\begin{aligned} \gamma (x, t) = \gamma _{\text {3D}}(x) + \gamma _{\text {4D}}(x, t) \end{aligned}$$

**Fig. 3:**
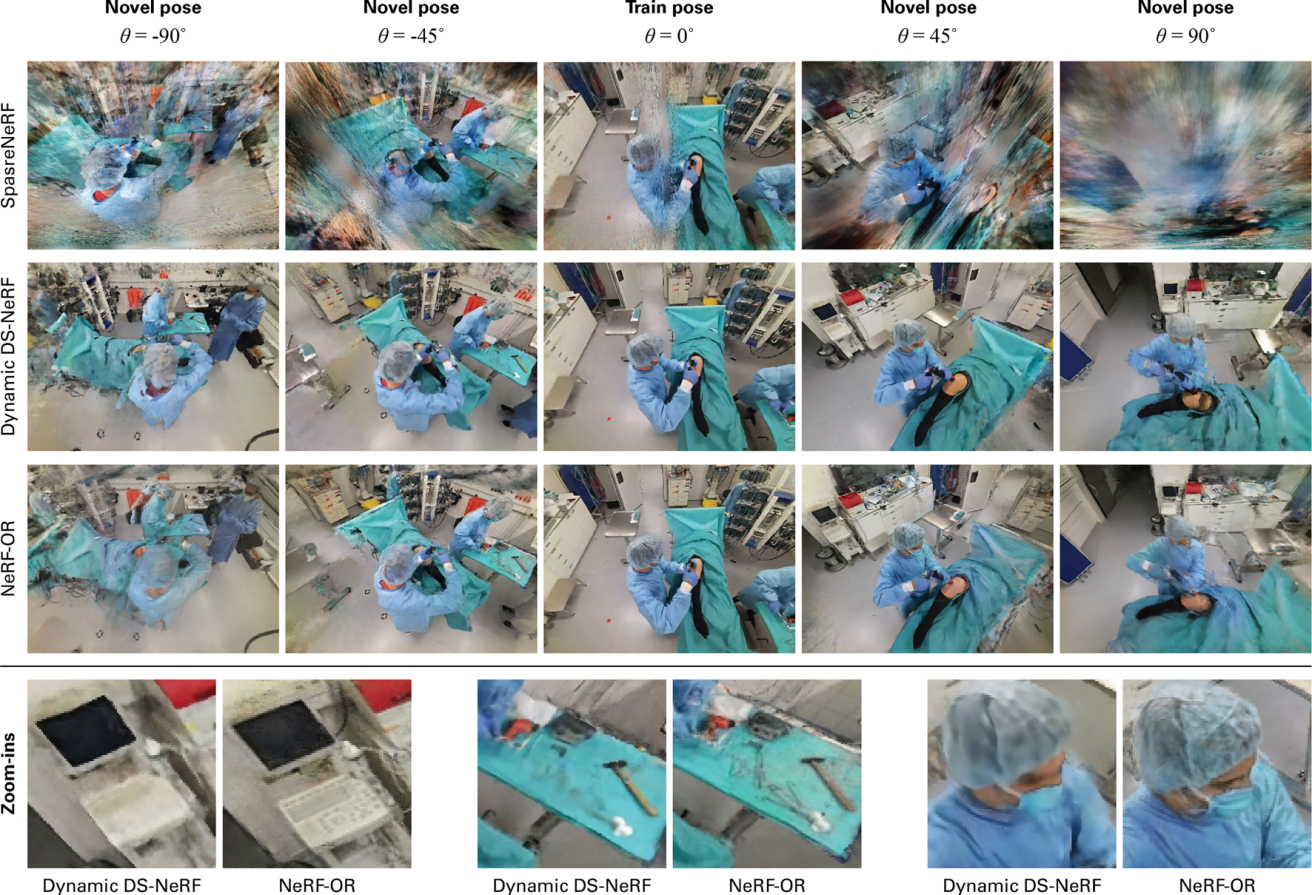
Qualitative comparison between SparseNeRF [22], Dynamic DS-NeRF [20], and NeRF-OR. Where SparseNeRF collapses to a sub-optimal solution, the other methods generate geometrically correct reconstructions. Below, three zoom-ins are presented of images synthesized by the latter two methods. In contrast to Dynamic DS-NeRF, the proposed method grasps fine details such as the keyboard, small instruments, or the surgeon’s face

### Datasets

We evaluate our method with two datasets. First, NeRF-OR is applied to dynamic scenes in the 4D-OR dataset [6] that display acted-out knee surgeries. This dataset consists of videos acquired by six fixed Azure Kinect RGB-D cameras spread throughout the OR, capturing the scene from very different perspectives. Camera locations and viewing directions are calculated with external calibration. Second, we use the NVS-RGBD benchmark dataset [22]. This benchmark includes eight static non-surgical scenes captured with a moving Azure Kinect RGB-D camera. Additional scenes are available where depth is recorded with the ZED 2 or iPhone LiDAR. However, we do not use these as the depth maps are not stored in absolute values or the scenes were not used for benchmarking earlier. Each scene consists of three randomly selected training images and various test images captured from unobserved camera angles. We assess image synthesis quality by comparison with test images in terms of PSNR, SSIM [29], and LPIPS [30].

### Implementation details

We train all NeRF-OR models with a patch size of $8\times 8$ pixels, $P_{\text {levels}}$ of 4, loss weights $\alpha$ and $\beta$ equal to 0.5, a batch size of 64 rays with 16,384 points sampled along each ray, and learning rate = 0.01. For the MLPs, we choose 3 layers with 256 neurons each. For the hash encoding, we choose: $N_{\text {min}} = 16$, $N_{\text {max}} = 2048$, and $T = 2^{21}$. When learning static scenes, NeRF-OR is trained for 2K iterations in 40 min on a single GPU. For dynamic scenes, we train for 10K iterations in 12 h on 4 GPUs. We use 48GB NVIDIA A40 GPUs and synthesize a single $640 \times 368$ image in 6 s. We use Marigold [23] as depth estimator, although other methods could be used as well.

## Results

### Qualitative evaluation on static surgical scenes

**Fig. 4:**
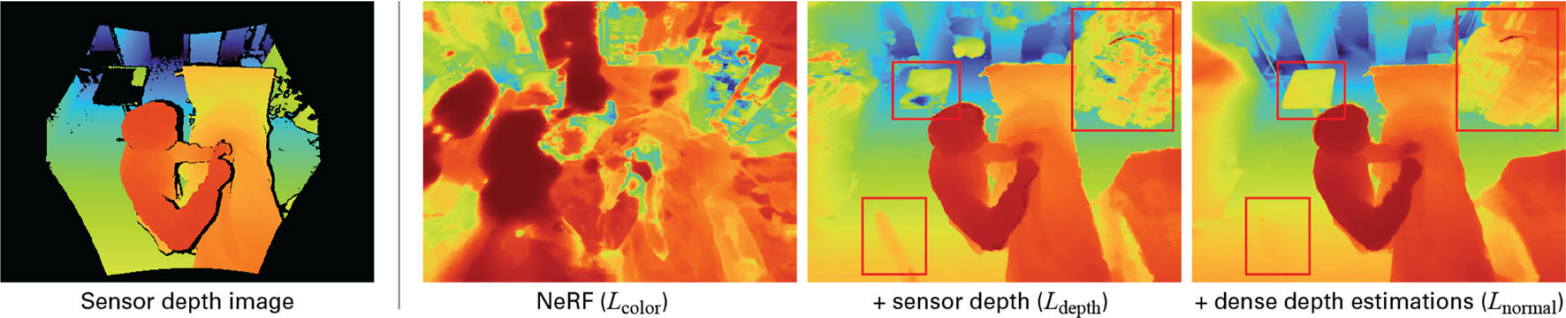
Left: example of sensor depth where black pixels mean no measurement. Right: predicted depth maps by NeRF-OR with loss function ablations. Red boxes indicate differences

**Table 1:** Comparison in render image quality for methods trained with five camera views and tested with a single unseen camera view in the 4D-OR dataset

		SparseNeRF [22]	DyDS-NeRF [20]	NeRF-OR (Ours)
Train time (min)		120	1200	40
Render time (sec)		14	22	6
Train	PSNR ($\uparrow$)	27.18 (± 0.12)	25.32 (± 0.26)	**29**.**90** (± 1.40)
views	SSIM ($\uparrow$)	0.818 (± 0.004)	0.817 (± 0.011)	**0**.**916** (± 0.022)
	LPIPS ($\downarrow$)	0.292 (± 0.006)	0.250 (± 0.017)	**0**.**163** (± 0.030)
Test	PSNR ($\uparrow$)	12.35 (± 0.16)	17.81 (± 0.33)	**18**.**15** (± 0.35)
view	SSIM ($\uparrow$)	0.260 (± 0.007)	0.514 (± 0.024)	**0**.**521** (± 0.024)
	LPIPS ($\downarrow$)	0.674 (± 0.010)	**0**.**487** (± 0.007)	0.492 (± 0.014)

Best performance is in bold

In a qualitative evaluation, we compare the reconstructions from NeRF-OR with those from SparseNeRF [22], which is state of the art for sparse-view datasets, and the earlier proposed Dynamic DS-NeRF [20]. The methods are trained on surgical scenes in the 4D-OR dataset using all six cameras as training views. Since SparseNeRF is a non-dynamic method, we train these methods on static scenes. Figure 3 shows the results for a scene with an ongoing surgical procedure. As evaluation, we render a circular camera motion around the surgical field, i.e., the operating table. The circular motion starts at a camera pose present in the training set (at angle $\theta = 0^\circ$). Then, we virtually move the camera around the circle, changing $\theta$. The figure shows that although SparseNeRF reconstructs the training pose mostly accurately, it displays heavy disruptions in images synthesized from novel views. Because the training cameras are too far apart, the method collapses to a sub-optimal solution that does not represent the scene geometry correctly. In contrast, Dynamic DS-NeRF and NeRF-OR are able to synthesize views without heavy disruptions from novel views. A limitation of the former method is the use of positional encoding, resulting in smoothed reconstructions that fail to grasp fine details. At the bottom of Fig. 3, we show that our method finds these details, e.g., in the keyboard, the small surgical instruments on the table, or in the surgeon’s face. The ability to capture high-frequency signals is due to the use of hash encoding.

That the geometric stability is a result of supervision with sensor depth and surface normals can be seen in Fig. 4. The figure shows the predicted depth maps by NeRF-OR for various sets of loss functions. When using color loss only, the method is not able to represent the scene accurately. The addition of depth loss is essential in finding correct representations. However, predicted depth values are noisy where the ToF sensor could not find any values. This can be seen at the table in the upper-left corner, the anesthetic machine in the upper-right corner, or the smoothness of the floor in the bottom-left. Additional supervision with surface normals corrects for these errors, resulting in the most accurate scene representations. More renders, of different scenes and camera angles, are given in Supplementary Materials.

### Quantitative evaluation on static surgical scenes

We quantify the image rendering quality by training NeRF-OR with five camera views and generating images for the sixth camera view unseen during training. The renders are of static scenes in all ten takes in the 4D-OR dataset, where we take the middle frame of each surgical take. The results are presented in Table 1, together with a comparison to SparseNeRF and Dynamic DS-NeRF. Due to hash encoding, NeRF-OR converges 3$\times$ faster than SparseNeRF and 30$\times$ faster than Dynamic DS-NeRF, while rendering times are reduced to 6 s.

Similar to our qualitative observations, SparseNeRF regenerates the training views accurately (PSNR of 27.18 dB) though it cannot synthesize accurate images from the unseen pose (12.35 dB). Dynamic DS-NeRF synthesizes less photorealistic images from training views due to overly smooth results. Therefore, the render quality from training views is reduced (25.32 dB), while the quality from an unseen pose is improved (17.81 dB). NeRF-OR gives the best balance between performance on train and test views, with a PSNR of 29.90 and 18.15 dB, respectively. The performance gap between train and test views is 11.75 dB, stressing the challenge of synthesizing images from camera views very different from those in the training set.

### Dynamic reconstructions of whole-procedure videos

**Fig. 5:**
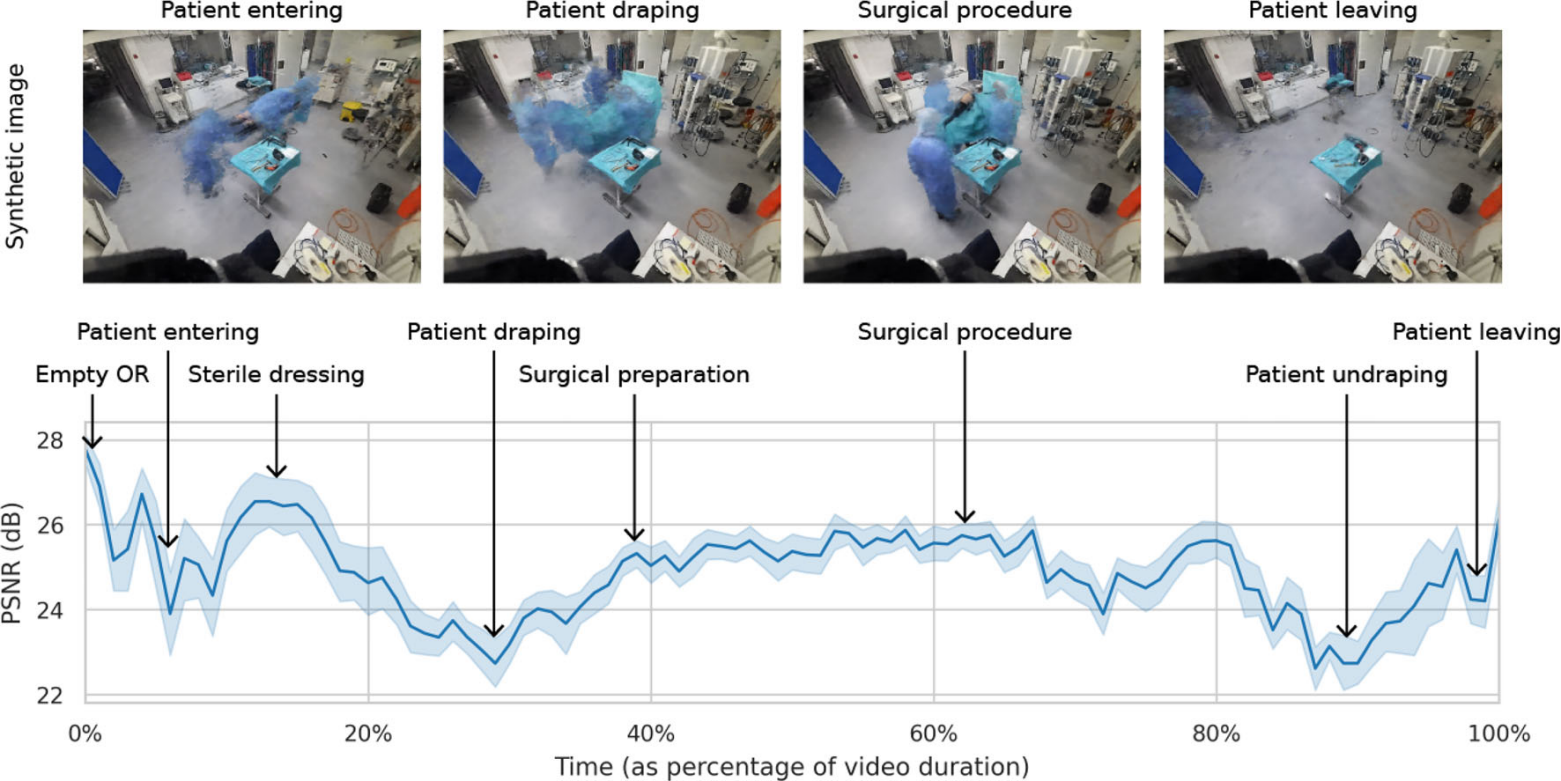
Dynamic renders of NeRF-OR fit to whole-surgery videos. Average PSNR is presented over time with various operative phases indicated. Example renders for four phases are given in the top row

For an evaluation of dynamic reconstructions, we fit NeRF-OR to whole-procedure videos in the 4D-OR dataset. For each video, we train a single NeRF to fit all uneven frame numbers in the video. Thereafter, we synthesize images at all even frames from the training view positions. In this way, we can assess the temporal quality when using our method for dynamic scenes. Figure 5 presents the average render quality as a function over the duration of the video. The image quality for each reconstruction ranges from a minimum PSNR of 17.53 dB to a maximum of 30.18 dB with an average of 24.86 dB (± 1.95). The image quality changes for various operative phases, with some phases typically resulting in increased quality (e.g., “empty OR,” “sterile dressing”) and others with reduced quality (e.g., “patient entering,” “patient draping”). These differences are explained by the amount of movement visible in the scene, where fast dynamic movements result in decreased image quality. It means that NeRF-OR can better represent static elements than dynamic elements, leaving room for further improvement. Nevertheless, our method can grasp coarse dynamic changes in the surgical videos, as can be seen in the example renders in Fig. 5.

**Table 2:** Image quality of rendered test images on Azure Kinect samples in the NVS-RGBD dataset

Method	PSNR $\uparrow$	SSIM $\uparrow$	LPIPS $\downarrow$
MonoSDF [21]	25.50	0.838	0.239
RegNeRF [18]	25.78	0.840	0.242
DS-NeRF [19]	25.91	0.838	0.238
SparseNeRF [22]	26.28	0.850	0.232
NeRF-OR (Ours)	**26**.**72**	**0**.**860**	**0**.**204**

Best performance is in bold

### NVS-RGBD sparse-view benchmark

Table 2 presents a comparison of NeRF-OR with state-of-the-art methods on the NVS-RGBD benchmark. Our method is able to reproduce test views with an average PSNR of 26.72 dB, improving upon existing methods for sparse-view datasets. This means that NeRF-OR can be applied in other sparse-view camera settings beyond the OR, such as the moving camera in this benchmark.

Table 3 presents an ablation study of NeRF-OR, where the depth and surface normals losses are enabled and disabled. As we have seen qualitatively for the 4D-OR dataset, the standard NeRF architecture cannot find accurate representations of the scene with too few training cameras. When supervised with ToF sensor depth, the render image quality drastically increases, with a PSNR from 19.07 to 26.37 dB. The additional supervision with surface normals results in more accurate color predictions, with a PSNR of 26.72 dB, although the difference in structural (SSIM) and perceptual (LPIPS) similarity is limited for this benchmark. The benefit of surface normals is only present in combination with a depth loss, as its isolated application results in low image quality.

**Table 3:** Ablation study: with and without sensor depth loss ($L_{\text {depth}}$) and surface normals loss ($L_{\text {normal}}$)

$L_{\text {depth}}$	$L_{\text {normal}}$	PSNR $\uparrow$	SSIM $\uparrow$	LPIPS $\downarrow$
✓	✓	**26**.**72**	**0**.**860**	0.204
✓	✗	26.37	0.858	**0**.**203**
✗	✓	16.77	0.624	0.506
✗	✗	19.07	0.721	0.338

Best performance is in bold

**Fig. 6:**
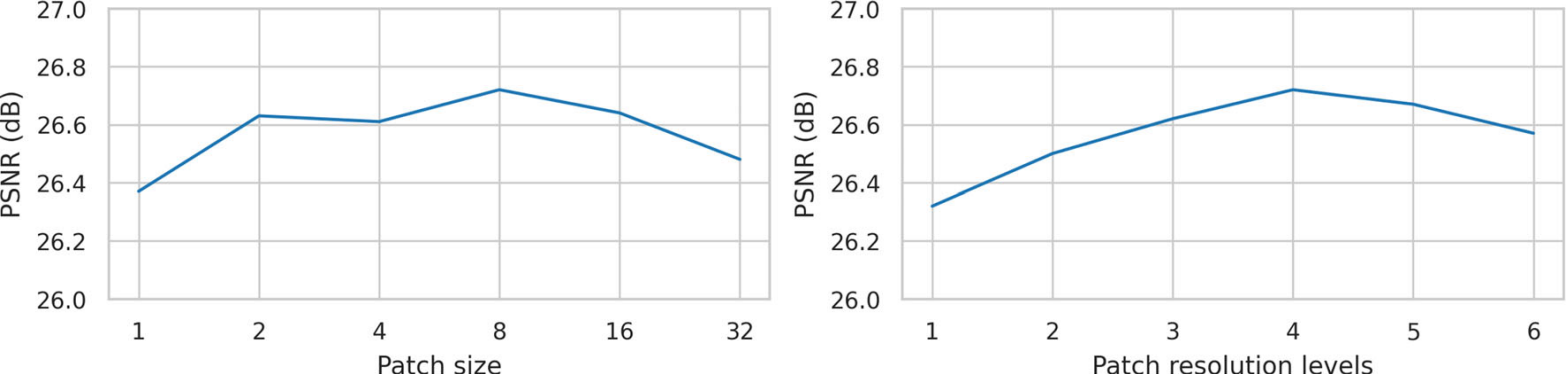
Image quality for various rectangular patch sizes and resolution levels $P_{\text {levels}}$. Note that a patch size of one is equal to NeRF-OR without surface normals regularization

NeRF-OR uses patch-based training such that surface normals can be calculated from horizontal and vertical depth gradients. We conducted an experiment in which we vary the patch size and patch resolution levels in the NVS-RGBD benchmark. Figure 6 shows that patches that are either too small or too large have a detrimental effect on performance, and the optimum appears to be a patch size of 8$\times$8 pixels. Likewise, we found that too few or too many resolution levels negatively affect results, and the optimum appears to be $P_{\text {levels}}=4$.

**Table 4:** Image quality when using different methods for dense depth estimation

Method	PSNR $\uparrow$	SSIM $\uparrow$	LPIPS $\downarrow$
DPT [26]	26.31	0.856	0.206
Depth Anything V2 [31]	26.55	**0**.**861**	0.207
Marigold [23]	**26**.**72**	0.860	**0**.**204**

Best performance is in bold

Table 4 lists NeRF-OR results for different dense depth estimators. The table displays a comparison between DPT [26], Depth Anything V2 [31] and Marigold [23], where the latter two are known to provide more accurate and fine-grained depth results. The results show that it is possible to use our framework with various monocular depth estimators. Potentially, the render quality could be further increased when even more accurate depth estimators are developed.

## Discussion

We presented NeRF-OR, a novel method for building neural representations of the operating room from a small set of RGB-D cameras. The method is designed for sparse datasets where the location and viewing direction of the training cameras differ heavily. We showed that the supervision with ToF sensor depth mitigates the challenging camera setup in the OR, which is in line with earlier research [20]. Nevertheless, these sensor depths result in noisy representations in areas where no depth values could be found. The missing values are the result of the sensor shape, reflective materials, and “depth shadows” around object boundaries. We found that additional supervision with surface normals increases the representation quality, especially in zero-valued sensor depth areas. The surface normals are calculated from dense depth maps gained with a monocular depth estimator from RGB images.

We showed the benefit of our approach qualitatively on surgical scenes in the 4D-OR dataset [6]. Where state of the art designed for sparse-view datasets collapsed to sub-optimal solutions, NeRF-OR was able to gain geometrically accurate representations. In contrast to our previous method [20], NeRF-OR grasps fine scene details while also training 30$\times$ faster. Quantitatively, our method finds the best balance between performance from the training views (PSNR of 29.90 dB) and unseen camera poses (18.15 dB). Considering this performance gap, image synthesis at camera angles very different from the training views remains challenging. It means that, when applying NeRF-OR, one needs to consider that rendering quality is high when close to the training views and decreases when traveling further away. With the use of 4D hash encoding, we were able to unite dynamic NeRFs with sparse-view training. We fit NeRF-OR to whole-procedure videos, where the method synthesized views at intermediate frame numbers with an average PSNR of 24.86 dB. We found that NeRF-OR performed better for phases with fewer movements (e.g., “empty OR,” “surgical procedure”) than in highly dynamic phases (e.g., “patient entering,” “patient draping”).

We benchmarked our method on the Kinect samples in the sparse-view NVS-RGBD dataset [22]. NeRF-OR renders novel views with an image quality of 26.72 PSNR, 0.860 SSIM, and 0.204 LPIPS, improving upon state of the art. This means that our method generalizes well to other few-camera setups beyond the OR. Nevertheless, it is important to validate the generalizability to other medical environments in the future. As the dataset that we used contains acted-out surgical scenes, it is particularly relevant to perform validations on real surgery datasets.

Over the last years, a large number of monocular depth estimators have been proposed [23, 25, 26, 32]. In our experiments, we showed that these estimators are directly applicable to images acquired in the OR, without the need for fine-tuning. Moreover, the quality of NeRF-OR image renders depends on the quality of the depth estimator. The development of new estimators that align better with the sensor depth could result in even better scene representations in the future.

While sensor depth helps to build geometrically correct representations, their use comes with a number of limitations as well. For example, inaccuracies during extrinsic calibration or errors in depth measurements can result in blurry textures or in objects positioned twice in the scene. Another issue arises at moving people or objects, caused by the asynchronicity of the depth measurements. Due to interference in the ToF mechanism of these sensors, it is impossible to capture all sensor depth images at exactly the same time. Mitigating these challenges would likely result in even sharper scene representations.

In the wake of NeRF, Gaussian Splatting [33] has been proposed as an alternative for novel view synthesis. The main advantage of this approach is a reduced time for image synthesis, enabling real-time rendering. Eventually, fast renders are essential to build interactive videos for educational or virtual reality purposes. For endoscopic video, Gaussian Splatting has been shown to achieve rendering speeds of up to 195 frames per second [34]. Similar to NeRF, Gaussian Splatting tends to over-fit in the sparse-view setting resulting in sub-optimal solutions and floating materials [35]. Recently, several sparse-view approaches were proposed for static scenes [35–38]. Future work should evaluate whether Gaussian Splatting is a valid alternative to NeRF for the challenging camera setups often found in clinical environments.

Other future work involves explorations to uses of NeRF-OR representations for downstream video processing tasks, such as 3D object detection [16] and segmentation [17]. Adapting the method to incorporate deformation fields [39] would make the method applicable for 3D tracking in the OR, which has been shown to be effective for laparoscopic videos already [12]. We believe that NeRF-OR can enable these future directions and we encourage other researchers to further explore the possibilities for neural fields in the OR.

## Supplementary Information

Below is the link to the electronic supplementary material.

Supplementary file 1 (pdf 9272 KB)

Supplementary file 2 (mp4 14578 KB)

Supplementary file 3 (mp4 26882 KB)

Supplementary file 4 (mp4 22888 KB)

Supplementary file 5 (mp4 38531 KB)
