# Supplementary material for: NeRF-OR: neural radiance fields for operating room scene reconstruction from sparse-view RGB-D videos
Source: Int J Comput Assist Radiol Surg. 2024 Sep 13;20(1):147–56. doi: 10.1007/s11548-024-03261-5 (PMC11758168; doi:10.1007/s11548-024-03261-5)
Supplement: Supplementary file 1 — (pdf 9272 KB) [file 11548_2024_3261_MOESM1_ESM.pdf]

Supplementary Materials

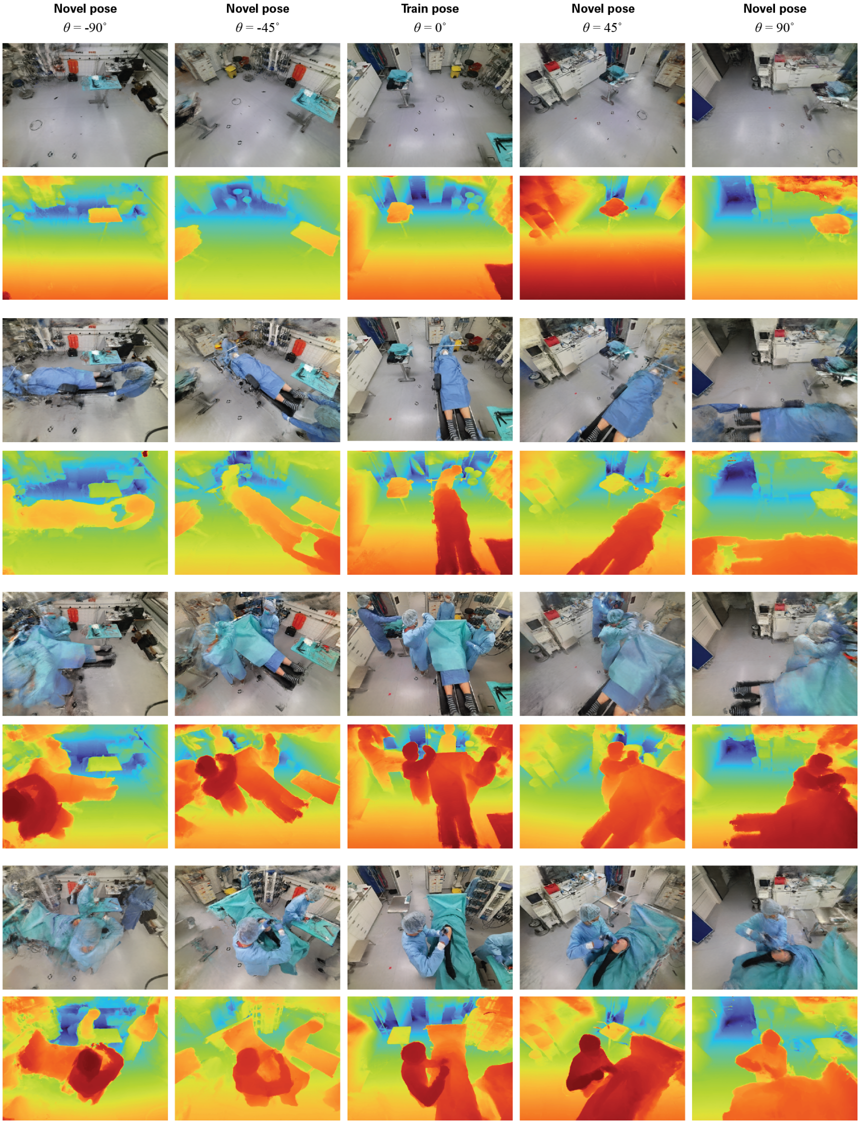

Fig. 7 Examples of renders with NeRF-OR on scenes in the 4D-OR dataset, including synthesized images and depth maps. Images in the middle column are rendered from a training pose. Other images are renders of a virtual camera surrounding the surgical field with an angle θ compared to the training pose.

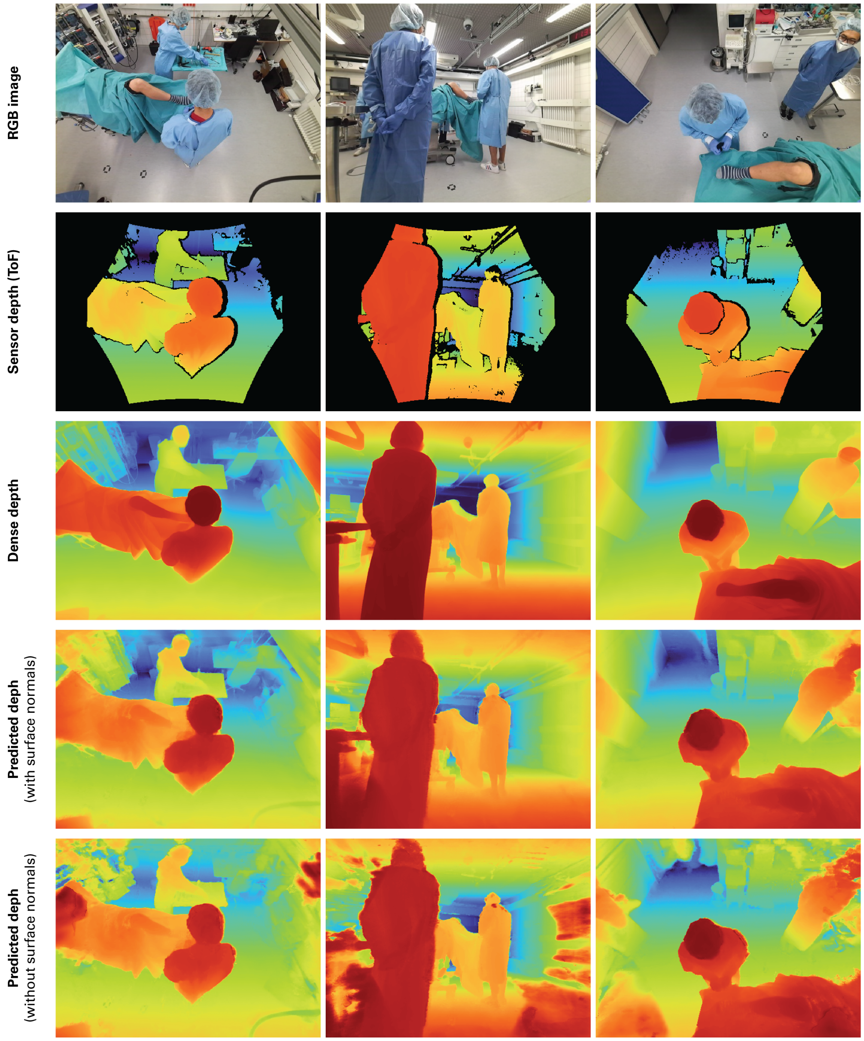

Fig. 8 Comparison of NeRF-OR scene reconstructions with and without surface normals loss (two bottom rows). The top rows display the input data during training: RGB, sensor depth and dense depth.
